# Metropolitan Hospital Sunday Fund

**Published:** 1903-01-10

**Authors:** 


### METROPOLITAN HOSPITAL SUNDAY FUND.

### A STRIKING DISPARITY.

THE Council meeting of this fund called for Tuesday afternoon (6th inst.) at the Mansion House was purely formal, the object being to elect committees and officers for the year. The Right Honourable the Lord Mayor presided, and there were about 25 members present, including Sir Edwin Durning Lawrence, Sir William Church, Sir William Broadbent, Canon Bristow, Canon McCormick, the Rev. Brook Deedes, Dr. Rigg, Mr. Thomas Bryant, Mr. John H. Hale, and the Secretary (Sir Edmund Hay Currie). Letters of apology had been received from Sir Sidney H. Waterlow, the Archdeacon of London, Lord Sandhurst, Prebendary Ridgeway, and others. The committees were all re-elected, with no alterations except that Mr. John H. Hale's name was added to the General Purposes Committee. Mr. Hale has been a valuable member of the Council for some years.

Sir Edmund Currie, speaking on the Special Committee for Surgical Appliance Orders and Hospital Letters, said he would be very glad if the "any two" members of the Council who by constitution act with the Secretary for this purpose would come to the office at any time when the applicants were seen; they would find the work very interesting, for applicants came from all over London. By the constitution five per cent. of the total collected was set apart for surgical appliances. The proportion of £60,000 (the record collection of 1902) was £3,000, and this sum brought in another £3,000 from the patients' payments. Last year 1,000 hospital letters were supplied. It was not often that the Council members put in an appearance.

The Lord Mayor, whose remarks were endorsed by the Secretary, drew attention to the striking disparity between the number of patients from country districts and the contributions from those districts to the Hospital Sunday Fund. The patients who come from outside the 12-mile radius bear a proportion of 25 per cent. to the whole number, while the number of contributing country districts is practically *nil.* This proportion, moreover, applies only to the larger hospitals; in the smaller ones it is still more striking, being something like 60 per cent. Of the 104,000 in-patient who annually benefit by the London hospitals, that is to say, 26,000 come from outside London. Out of £600,000, the annual cost of London's in-patients, £150,000 is spent on these country patients. The country clergy plead that they cannot help much on account of their own cottage hospitals; but the disparity is one that requires, to quote Sir Edmund Hay Currie, "some more direct appeal."

A special meeting will be held at the Mansion House on the Monday preceding Hospital Sunday (June 8th), which, it is hoped, will be widely reported. It will probably be addressed by some eloquent speaker, and good results are looked for.

